# Recurrent Femoral Osseous Hydatid Disease Managed With Repeated Intramedullary Curettage: A Case Report

**DOI:** 10.7759/cureus.109815

**Published:** 2026-05-28

**Authors:** Khatab Zakaria, Lamzira Mohammed, Achraf Tebbaa El Hassali, Najib Abdeljaouad, Hicham Yacoubi

**Affiliations:** 1 Department of Orthopedics and Traumatology, Centre Hospitalier Universitaire Mohammed VI, Oujda, MAR; 2 Department of Orthopedic Surgery, Centre Hospitalier Universitaire Mohammed VI, Oujda, MAR; 3 Department of Orthopedics and Traumatology, Mohammed VI University Hospital, Oujda, MAR; 4 Faculty of Medicine and Pharmacy, Mohammed First University, Oujda, MAR

**Keywords:** echinococcosis, femur, hydatid disease, orthopedic surgery, osseous hydatidosis, recurrence

## Abstract

Hydatid disease is a parasitic infection primarily caused by *Echinococcus granulosus*. Osseous hydatid disease is an uncommon manifestation and may present significant diagnostic and therapeutic challenges because of its insidious progression and tumor-like appearance. Femoral involvement is particularly rare. We report the case of a 44-year-old hypertensive woman who presented with chronic mechanical pain of the right hip evolving since 2019 and initially treated as lumbosciatica without improvement. MRI revealed an extensive intramedullary osteolytic lesion involving the right femoral diaphysis over 25 cm, initially suggestive of a bone tumor. Histopathological examination following femoral biopsy confirmed intraosseous hydatid disease. The patient underwent intramedullary curettage, reaming, and scolicidal irrigation associated with prolonged albendazole therapy. Despite initial treatment, MRI follow-up demonstrated local recurrence requiring revision surgery with repeated intramedullary debridement and irrigation using hydrogen peroxide and hypertonic saline. At more than six months of follow-up after revision surgery, the patient remained pain-free with preserved limb function and no pathological fracture. This case highlights the diagnostic difficulties, high recurrence potential, and therapeutic challenges of femoral osseous hydatidosis. In endemic regions, hydatid disease should be considered in the differential diagnosis of chronic osteolytic lesions of long bones.

## Introduction

Hydatid disease is a zoonotic parasitic infestation caused mainly by *Echinococcus granulosus* and remains endemic in many Mediterranean, Middle Eastern, African, and South American countries [1,2]. The liver and lungs are the most frequently involved organs, whereas osseous localization is uncommon and represents only a small proportion of all hydatid localizations [1,3]. Among skeletal localizations, the spine and pelvis are the most commonly affected sites, while isolated femoral involvement remains particularly rare [3,4].

Osseous involvement is believed to result from hematogenous dissemination of the parasite to bone tissue. Unlike visceral hydatid cysts, osseous hydatidosis lacks a true pericyst, allowing progressive intramedullary extension along trabecular bone channels [2]. This infiltrative growth pattern contributes to delayed diagnosis, extensive bone destruction, and a high risk of recurrence [3,4]. Clinical manifestations are often nonspecific and may include chronic pain, swelling, pathological fractures, or functional impairment [4]. Depending on lesion location, symptoms may be misleading and can mimic other musculoskeletal or radicular conditions, including sciatica, which may delay diagnosis. Radiological findings frequently mimic primary bone tumors, chronic osteomyelitis, or metastatic lesions, making diagnosis challenging [5].

MRI is considered the imaging modality of choice for evaluating lesion extension, cortical involvement, and soft tissue extension, whereas definitive diagnosis relies on histopathological examination [5,6]. Therapeutic management remains difficult because complete surgical excision is frequently impossible in long bones, especially in extensive lesions [4,7]. Recurrence remains common despite combined surgical and antiparasitic treatment [4,7].

We report a rare case of recurrent intraosseous hydatid disease involving the right femoral diaphysis in a 44-year-old woman treated with repeated intramedullary curettage, reaming, and scolicidal irrigation associated with prolonged albendazole therapy. This case highlights the diagnostic difficulties and therapeutic challenges associated with femoral osseous hydatidosis.

## Case presentation

A 44-year-old woman with a past medical history of hypertension presented with chronic right hip pain evolving over several years. The pain was mechanical in nature. She initially consulted several physicians and received symptomatic treatment for presumed lumbosciatica without clinical improvement. Because of persistent symptoms, she later consulted a private orthopedic surgeon, who requested an MRI. Based on the imaging findings, a biopsy was performed, confirming osseous hydatid disease, and the patient was subsequently referred to our department for further management. There was no history of trauma, fever, weight loss, or constitutional symptoms. The patient was from an endemic area for hydatid disease, with no specific history of animal contact reported.

Routine laboratory investigations showed no peripheral eosinophilia. C-reactive protein was elevated. No *Echinococcus *serological testing was performed. Radiographs of the right femur demonstrated multiple osteolytic intramedullary lesions involving the femoral diaphysis with cortical thinning. MRI revealed an extensive centromedullary osteolytic process involving the right femoral shaft over approximately 25 cm, measuring 30 × 21 mm in transverse diameter, associated with cortical remodeling and multiple cystic formations suggestive of a tumoral lesion (Figure 1, Figure 2).

**Figure 1:**
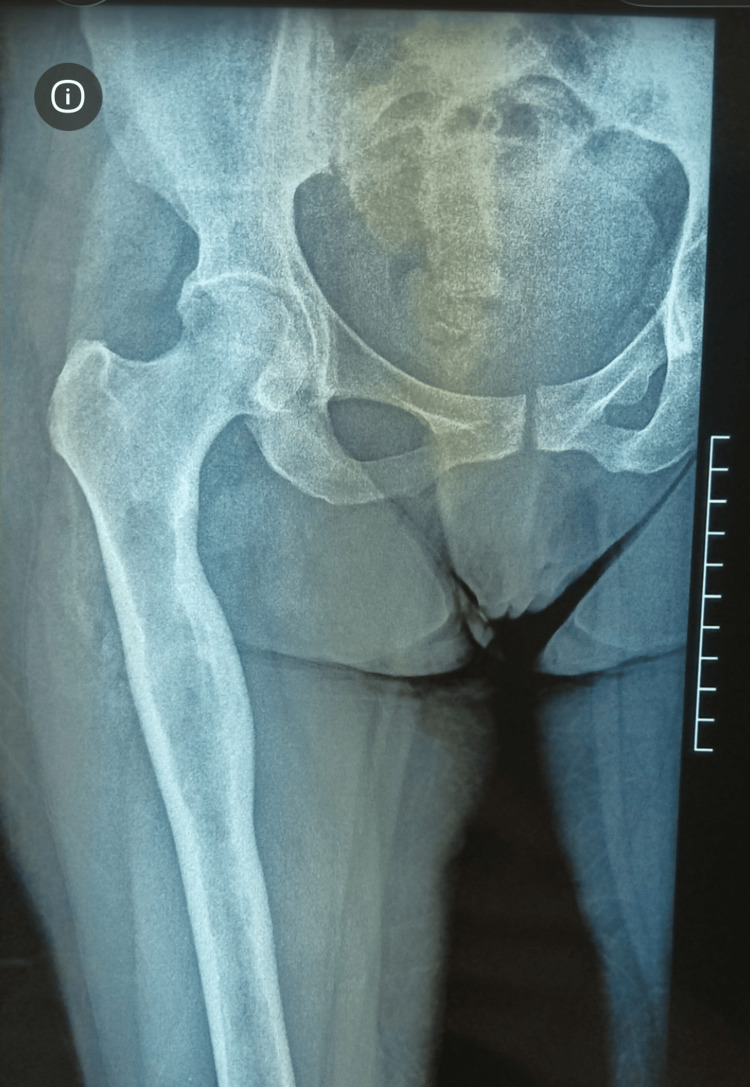
Plain radiograph of the right femur showing extensive multiloculated osteolytic intramedullary lesions involving the femoral diaphysis with cortical thinning

**Figure 2:**
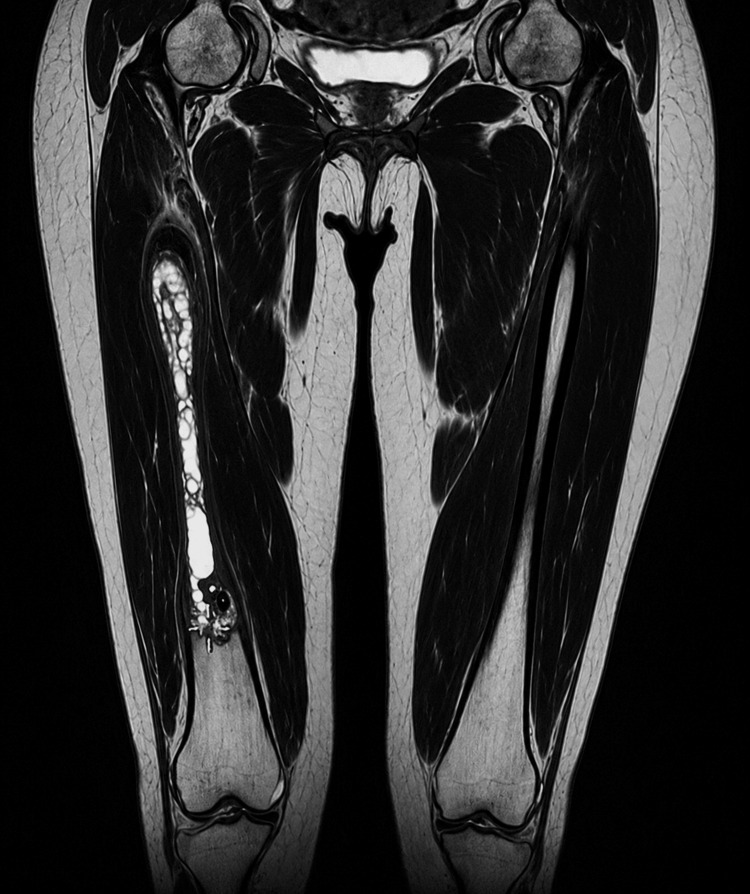
Coronal MRI view showing elongated intramedullary multicystic lesions involving the right femoral diaphysis

Histopathological examination of the femoral biopsy demonstrated hydatid membranes composed of laminated cuticle and germinal membrane, consistent with intraosseous hydatid disease. Extension workup, including thoracic CT, showed no additional visceral localization.

Albendazole therapy was initiated after histopathological confirmation of the diagnosis at a dose of 400 mg twice daily and was continued for nine months. Surgical management was then performed through a subtrochanteric approach and a distal femoral cortical window. Intramedullary curettage and progressive reaming of the femoral canal were carried out. Intraoperative irrigation was performed using diluted hydrogen peroxide and hypertonic saline as scolicidal agents. Histopathological analysis of the operative specimen confirmed hydatid disease without evidence of malignancy.

Follow-up MRI revealed recurrence within the proximal third of the femoral medullary cavity, characterized by a lobulated cystic lesion measuring 15 × 52 mm, hypointense on T1-weighted images and hyperintense on T2-weighted images, associated with endosteal erosion and cortical thickening without soft-tissue invasion (Figure 3).

**Figure 3:**
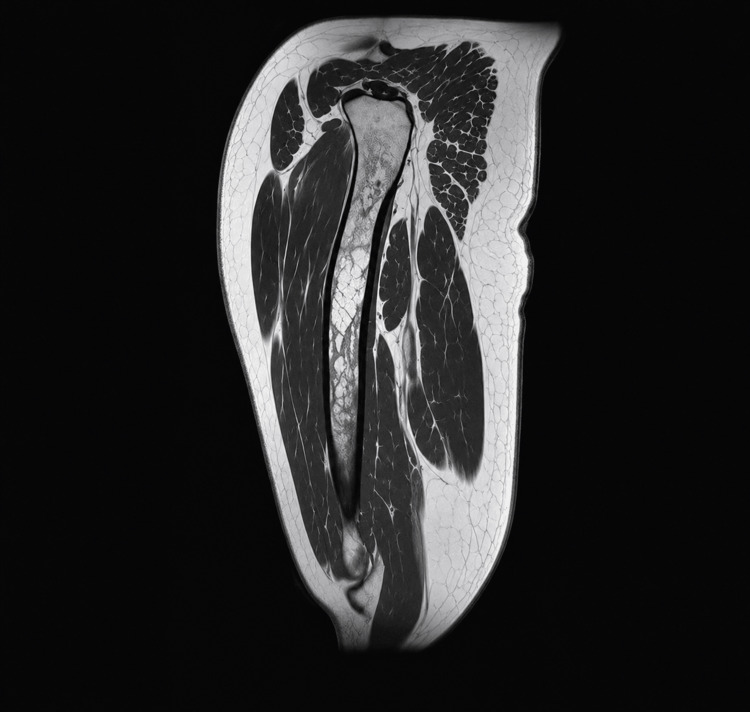
Follow-up MRI demonstrating recurrent lobulated cystic lesion within the femoral medullary cavity associated with endosteal erosion and cortical thickening

Revision surgery was subsequently performed. The patient underwent repeat intramedullary reaming and debridement through dual surgical approaches, with extensive lavage using hydrogen peroxide and hypertonic saline. Bacteriological and pathological samples were obtained intraoperatively. Intraoperative photographs illustrated the proximal and distal surgical approaches and the intramedullary irrigation system used during revision surgery (Figure 4).

**Figure 4:**
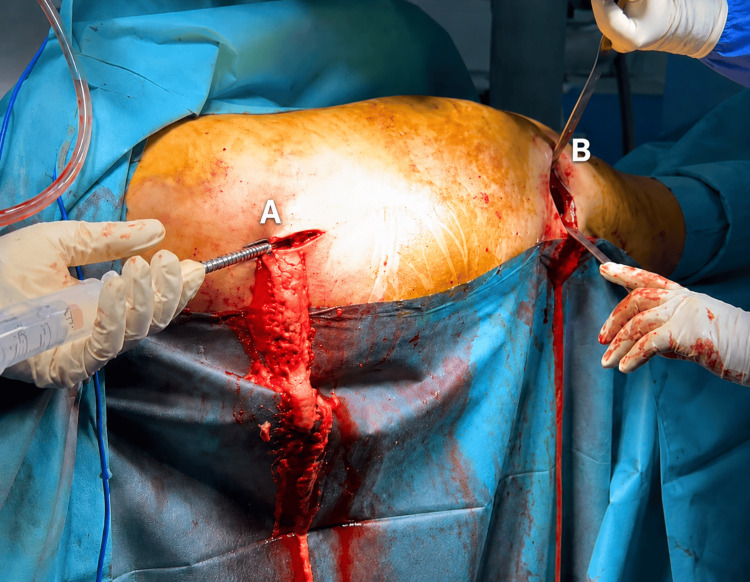
Intraoperative photograph showing (A) the proximal surgical entry point and (B) the distal cortical approach used for intramedullary curettage and irrigation during revision surgery

At more than six months of follow-up after revision surgery, the patient remained asymptomatic, with complete pain relief, preserved walking ability, no limping, no functional limitation, and no evidence of pathological fracture.

## Discussion

Bone hydatid disease is an uncommon form of echinococcosis and represents a small proportion of all hydatid localizations [1,3]. Skeletal involvement predominantly affects the spine and pelvis, while isolated femoral localization is rarely reported in the literature [3,4]. The absence of a limiting pericyst within bone tissue allows the parasite to spread progressively through the medullary canal and trabecular structures, resulting in delayed diagnosis and extensive local destruction [2].

The clinical presentation is often misleading because symptoms are nonspecific and slowly progressive. Patients may present with pain, swelling, limping, or pathological fracture depending on the stage of the disease [4]. Depending on lesion location, symptoms may mimic other musculoskeletal or radicular conditions, which can delay diagnosis. In the present case, the patient presented with chronic pain initially managed as lumbosciatica, contributing to delayed recognition of the underlying bone lesion. This illustrates the diagnostic difficulty of osseous hydatid disease and the importance of maintaining a broad differential diagnosis in patients with persistent, unexplained symptoms. At the latest follow-up, the patient had satisfactory functional recovery with preserved gait, absence of limping, and no evidence of pathological fracture.

Radiological investigations are essential for evaluating lesion extent and planning surgical management. MRI is considered the imaging modality of choice because it accurately assesses intramedullary extension, cortical damage, and possible soft-tissue involvement [5,6]. In our patient, MRI revealed multiloculated cystic intramedullary lesions associated with cortical thinning and endosteal erosion without periosteal reaction or neurovascular extension. However, these radiological findings are not pathognomonic and may mimic chronic osteomyelitis or primary bone tumors [5].

Definitive diagnosis relies on histopathological examination. In this case, both the initial biopsy and the postoperative specimens demonstrated hydatid membranes and laminated cyst wall structures consistent with osseous echinococcosis while excluding malignant disease. Routine laboratory investigations showed no peripheral eosinophilia, while inflammatory markers were elevated. No serological testing for *Echinococcus *was performed. Extension workup, including thoracic CT, did not reveal secondary localization.

Management of bone hydatidosis remains difficult because complete eradication of the parasite is challenging [4,7]. Surgical treatment remains the main therapeutic option and generally consists of aggressive curettage associated with evacuation of hydatid vesicles and extensive irrigation using scolicidal agents [4,7]. In our case, the lesion was approached through two cortical windows, allowing evacuation of multiple cystic components and intramedullary curettage (Figure 4).

Adjunctive antiparasitic therapy plays an important role in reducing recurrence risk. Albendazole is commonly administered before and after surgery, particularly in extensive osseous involvement [7]. However, there is no universally accepted duration of treatment in osseous hydatid disease. Our patient received albendazole therapy for nine months, with a favorable clinical evolution.

Despite combined surgical and medical treatment, recurrence remains frequent in osseous hydatid disease because of microscopic bone infiltration [4,7]. Long-term radiological and clinical surveillance is therefore mandatory. This observation emphasizes the importance of considering hydatid disease in the differential diagnosis of atypical cystic bone lesions, especially in endemic regions, to improve early recognition and management.

## Conclusions

Primary hydatid disease of the femur is a rare entity that may present significant diagnostic and therapeutic challenges because of its insidious progression and nonspecific clinical and radiological features. This case highlights the importance of considering osseous hydatidosis in the differential diagnosis of cystic or osteolytic bone lesions, particularly in endemic regions. MRI and histopathological examination remain essential for accurate diagnosis and evaluation of lesion extent. Combined surgical management and prolonged antiparasitic therapy can provide satisfactory functional outcomes despite the known risk of recurrence. Long-term clinical and radiological follow-up remains mandatory in order to detect possible recurrence at an early stage.
